# *Salmonella* antimicrobials inherited and the non-inherited resistance: mechanisms and alternative therapeutic strategies

**DOI:** 10.3389/fmicb.2023.1176317

**Published:** 2023-05-25

**Authors:** Kaixiang Zhou, Lei Sun, Xuehua Zhang, Xiangyue Xu, Kun Mi, Wenjin Ma, Lan Zhang, Lingli Huang

**Affiliations:** ^1^Department of Veterinary Medicine Science, College of Veterinary Medicine, Huazhong Agricultural University, Wuhan, Hubei, China; ^2^National Reference Laboratory of Veterinary Drug Residues (HZAU), Wuhan, Hubei, China; ^3^MOA Key Laboratory for Detection of Veterinary Drug Residues, Wuhan, Hubei, China

**Keywords:** *Salmonella*, resistance, tolerance, out membrane, probiotics, *predatory bacteria*, clinical application

## Abstract

*Salmonella* spp. is one of the most important foodborne pathogens. Typhoid fever and enteritis caused by *Salmonella enterica* are associated with 16–33 million infections and 500,000 to 600,000 deaths annually worldwide. The eradication of *Salmonella* is becoming increasingly difficult because of its remarkable capacity to counter antimicrobial agents. In addition to the intrinsic and acquired resistance of *Salmonella*, increasing studies indicated that its non-inherited resistance, which commonly mentioned as biofilms and persister cells, plays a critical role in refractory infections and resistance evolution. These remind the urgent demand for new therapeutic strategies against *Salmonella*. This review starts with escape mechanisms of *Salmonella* against antimicrobial agents, with particular emphasis on the roles of the non-inherited resistance in antibiotic failure and resistance evolution. Then, drug design or therapeutic strategies that show impressive effects in overcoming *Salmonella* resistance and tolerance are summarized completely, such as overcoming the barrier of outer membrane by targeting MlaABC system, reducing persister cells by limiting hydrogen sulfide, and applying probiotics or predatory bacteria. Meanwhile, according to the clinical practice, the advantages and disadvantages of above strategies are discussed. Finally, we further analyze how to deal with this tricky problems, thus can promote above novel strategies to be applied in the clinic as soon as possible. We believed that this review will be helpful in understanding the relationships between tolerance phenotype and resistance of *Salmonella* as well as the efficient control of antibiotic resistance.

## 1. Introduction

*Salmonella* spp. is gram-negative, facultative anaerobes and facultative intracellular bacteria that are divided into *Salmonella enterica* (*S. enterica*) and *Salmonella bongori* (Ryan et al., 2017). *S. enterica* is estimated to cause 16–33 million infectious cases, with 500,000–600,000 annual deaths worldwide (Bula-Rudas et al., 2015). Reportedly, the confirmed number of deaths was 94,530 in the European Union in 2016 (Chlebicz and Śliżewska, 2018). 50% of US swine operations were fecal positive for *Salmonella* (Bearson, 2022), and the economic losses due to salmonellosis in America are estimated to exceed $ 3.5 billion annually (Kim and Isaacson, 2017). More seriously, in many African countries, the incidence of typhoid fever is above 1/1000 per year, with approximately 1% of patients’ dying (Crump and Heyderman, 2015).

Infections caused by *Salmonella* may be due to direct contact with infected animals and or indirect contact via their environment. In addition, *Salmonella* in animal intestines can transfer onto their products due to careless processing or improper hygiene (Yeh et al., 2017). Therefore, animal-derived products are an important media for *Salmonella* spread and infections. According to the reports, *Salmonella* causes over 90 million diarrhea-associated diseases annually worldwide, 85% of which are linked to animal food consumption (Huang et al., 2018). Obviously, the animal-derived products and food packaging are one of the main reasons of the salmonellosis spread.

What is more worrying is that the antibiotic resistance of *Salmonella* is increasing over time. Zeng et al. (2019) reported that 92 *Salmonella* strains isolated from 672 samples in different provinces of China showed resistance to one antimicrobial agent at least. Reportedly, the resistance of 124,347 *Salmonella* isolates that were reported from 1990 to 2018 worsened for all antimicrobials in all regions (Browne et al., 2020). According to the National Animal Health Monitoring System, 20% of *Salmonella* from swine are multidrug-resistant (resistant to ≥3 antimicrobial classes) (Bearson, 2022). Facing the present serious resistance situation, the World Health Organization (WHO) included *Salmonella* on the priority list for the development of new antimicrobials in 2017 (WHO, 2017). On the contrary, increasing studies indicated that the non-inherited resistance (e.g., persister cells, biofilms) of *Salmonella* plays a critical role in the resistant evolution and antibiotic failure. For instance, antibiotics were evidenced to show poor activity against slowly replicating *Salmonella* in mice (Claudi et al., 2014). In addition, *Salmonella* persister cells that were reported could enhance the spread of resistance plasmids in the host gut (Bakkeren et al., 2019).

However, the development of new antibiotics has entered a bottleneck period. To solve the challenge of increasing resistance in *Salmonella*, the use of old drugs to develop new treatment strategies is more feasible. Meanwhile, history has been shown that antibiotic resistance always occurs, which reminded that more attention has to be paid on understanding the resistant evolution processes of *Salmonella*. Therefore, this review starts from the combatting methods of *Salmonella* against antimicrobial agents, including asymmetrical outer membrane, efflux pump, and the non-inherited resistance according to the newest studies, thus to completely understand its escape mechanisms to antimicrobial agents. On this basis, the novel treatment strategies show potential in countering *Salmonella* resistance, such as enhancing the permeation of outer membrane (OM) by interfering MlaABC system (the phospholipid transport system maintains the asymmetry of OM), reducing persister cells by inhibition the hydrogen sulfide (H_2_S) producing protein, and using *predatory bacteria* as the biology disinfection, which are summarized. We emphasize that this review will provide new perspectives and ideas for the prevention and control of the current severe health concerns caused by *Salmonella*.

## 2. Resistance mechanisms of *Salmonella*

### 2.1. Intrinsic resistance

#### 2.1.1. Asymmetrical outer membrane

**Figure 1:**
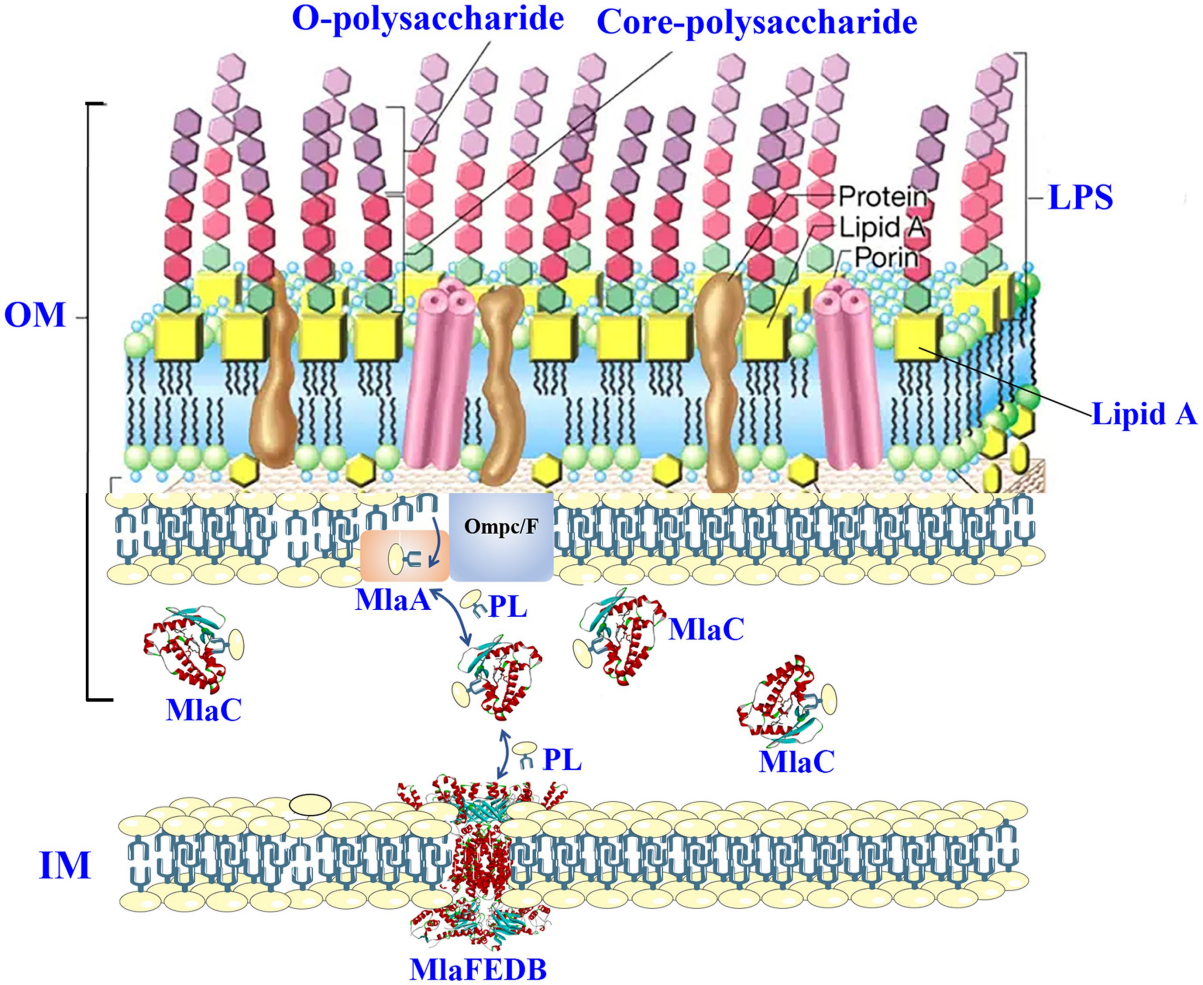
MlaABC transport system maintains the OM asymmetrical of gram-negative bacteria. LPS, lipopolysaccharide; PL, phospholipid; MlaC, PDB ID 7VR6; MlaFEDB, PDB ID 6Z5U.

As a gram-negative bacterium, *Salmonella* possesses a thick cell wall that consists of an OM, an inner membrane (IM), and a thin peptidoglycan layer, thus forming the periplasmic space (Figure 1; Pang et al., 2019). The asymmetrical outer membrane is believed to be involved in combatting antimicrobial agents. For instance, Wu et al. (2015) augured that the presence of the hydrophilic carbohydrate components of lipopolysaccharide (LPS) will form hydrated spheres, thereby limiting the movement and permeability of hydrophobic molecules on *Salmonella* cell membrane. At the same time, because the molecular structure of lipid A and the content of unsaturated fatty acids are lower than those of normal phospholipid bilayers, the effective packaging of lipids reduces the fluidity of the OM (Piggot et al., 2011; Khalid et al., 2015), thus limiting the permeation of hydrophobic antibiotic agents through the OM. Vancomycin, the glycopeptide class, is a classic example that is limited by OM. Vancomycin is an inhibitor of the peptidoglycan cross-linking, which is effective in gram-positive bacteria rather than in gram-negative bacteria due to the lack of permeation through the OM (Tsuchido and Takano, 1988).

Increasing studies proved that the asymmetrical OM is maintained by the MlaABC system, which removes and traffics phospholipids found in the outer leaflet of the OM to the IM (Figure 1; Ekiert et al., 2017; Hughes et al., 2019). These reminded that inhibitors aiming at MlaABC system of *Salmonella* are likely to be the potential antibiotic enhancers. Meanwhile, the agents targeting the components of OM, such as targeting phosphatidylglycerol or LPS, could disturb the asymmetrical structure, thus showing potency in improving antibacterial effects of existing antibiotics against multiple resistance *Salmonella* (Song et al., 2020).

#### 2.1.2. Efflux pumps

To date, reports have shown that *Salmonella* expresses nine kinds of efflux pumps, namely AcrAB, AcrEF, AcrD, MdsABC, MdtABC, EmrAB, MdfA, MacAB, and MdtK (Table 1; Yamagishi et al., 2011; Sun et al., 2014; Prajapati et al., 2021). Among them, AcrAB is known to be responsible for fluoroquinolone resistance (Giraud et al., 2000; Shen et al., 2017). For instance, in our previous studies, we induced the *Salmonella* mutation SI3 with a high concentration of ciprofloxacin. Further studies indicated that *ramA* played a key role in increasing the overexpression level of the AcrAB efflux pump (Sun et al., 2011). In addition, AcrAB functions in *β*-lactam resistance (Giraud et al., 2000). EmrAB and AcrEF contribute to glycylcycline, triclosan, and tigecycline resistance (Rensch et al., 2014). MacAB is responsible for the efflux of macrolides and plays an important role in antioxidant stress (Yamagishi et al., 2011; Bogomolnaya et al., 2013). Song et al. (2014) reported that the overexpression of the MdsABC pump in *Salmonella* indicates neomycin, crystal violet, rhodamine, and gold resistance. Metal resistance is mediated by AcrD and MdtABC (Nishino et al., 2007). In addition to expelling antimicrobial compounds out of bacterial cells, efflux pumps also contribute to biofilm formation and quorum sensing (Yang et al., 2006). Baugh et al. (2012) suggested that the presence of the efflux genes *acrD*, *emrAB*, *acrEF*, *acrB*, *macAB*, *mdsABC*, *mdfA*, *mdtK*, *mdtABC*, and *tolC* resulted in the induction of biofilm formation compared with the wild-type strain.

**Table 1:** A summary of the *Salmonella* efflux pumps and their substrates.

Efflux pump	Substrate	Regulator	Reference
AcrAB	Fluoroquinolones, tetracyclines	*AcrR, MarA, RmaA, RmaR, SoxS*	Shen et al. (2017)
AcrEF	Glycidylcyclines and tigecycline	*/*	Rensch et al. (2014)
EmrAB	Glycidylcyclines and tigecycline	*GolS*	Rensch et al. (2014)
MacAB	MacAB	MacAB	Yamagishi et al. (2011)
MdsABC	Neomycin, crystal violet, gold	*CpxAR*	Song et al. (2014)
AcrD	Copper, zinc	*CpxAR, AcrS*	Nishino et al. (2007)

#### 2.1.3. Antibiotic-inactivating enzymes

The antibiotic-inactivating enzymes produced by *Salmonella* can decompose or modify antibiotics. For instance, *Salmonella* resistance to *β*-lactam antibiotics is commonly the result of the production of *β*-lactamases that are encoded by blaTEM genes. *β*-lactamases can break the amide bond of the *β*-lactam ring, thus leading to inactivation of *β*-lactam antibiotics (Wright, 2005). Because cephalosporins are commonly used to treat *Salmonella* infections in the clinic, *β*-lactamase plays a key role in the resistance of *Salmonella*. For instance, the *β*-lactamases produced by *Salmonella* strains show resistance to ceftriaxone, ceftiofur, cefoxitin, ampicillin, and amoxicillin/clavulanic acid (Coculescu et al., 2014). Wu et al. (2015) isolated 60 *Salmonella* strains that produced *β*-lactamases from 699 foodborne samples, in which the *β*-lactamase-encoding gene *bla* (TEM-1) was observed most (n = 44), followed by *bla* (OXA-1) (*n* = 38).

Aminoglycosides contain an aminocyclitol parent ring that links to amino sugars by glycosidic bonds. Aminoglycoside resistance is commonly associated with three types of aminoglycoside-modifying enzymes, including acetyltransferases (AACs), adenyltransferases (ANTs), and phosphotransferases (APHs) (Mąka and Popowska, 2016). Reportedly, in 10 *Salmonella* isolates with aminoglycosides resistance, the aminoglycoside-modified enzyme ANT (2″) was found (Leegaard et al., 1996).

### 2.2. Acquired resistance

#### 2.2.1. Acquired resistance by mutations

Acquired resistance by mutations refers to *Salmonella* producing antibiotic resistance genes, which can be caused by base mismatch during replication. Base mismatch has a certain probability, though very small it occurs. Mutation in resistance genes may induce the overexpression of proteins (e.g., efflux pumps and antibiotic-inactivating enzymes), thus reducing antibiotic uptake or causing modifications to antibiotic targets (Uddin and Ahn, 2018; Pang et al., 2019).

In *Salmonella*, quinolone resistance was partly attributed to a point mutation in the *gyrA* gene encoding the gyrase subunit. The complex of gyrase and DNA is the main target of quinolones. Resistance mutations of the *gyrA* cluster in a region of the gene product between amino acids 67 and 106, called the quinolone resistance-determining region (QRDR). Hopkins et al. (2005) reported that amino acid changes at Ser-83 (change to Phe, Tyr, or Ala) or at Asp-87 (change to Gly, Asn, or Tyr) could be frequently observed in nalidixic acid-resistant *Salmonella* strains. In addition, an *in vitro* dynamic model indicated that the resistance of *Salmonella* against difloxacin, enrofloxacin, and marbofloxacin was targeted by a mutation in *gyrA* (S83F) (Lee et al., 2017).

Unlike the export action of efflux pumps, *Salmonella* protein channels (e.g., OmpC/F) located in the OM form an entry route for many antibiotics into cytosol. It was evidenced that OmpC and OmpF of *Salmonella* were responsible to the translocating of carbapenems, chloramphenicol, and cephalosporins, respectively (Medeiros et al., 1987; Armand-Lefèvre et al., 2003). That is why the changes in OmpC and OmpF, such as downregulation, mutations in the interior region, or expression of alternative porins, will develop resistance. For instance, loss of OmpC porin in *Salmonella typhimurium* causes increased resistance to cephalosporins (Medeiros et al., 1987).

#### 2.2.2. Acquiring resistance genes

In addition to gene mutation, antibiotic resistance genes can be obtained from the external environment. Antibiotic resistance genes can be carried on plasmids, transposons, integrons, and prophages, and bacteria can acquire these genes via horizontal gene transfer from bacterial species and phages (Pang et al., 2019). Reportedly, Alcaine et al. (2005) proved that the isolated ceftiofur-resistant *Salmonella* strains in dairy farms evolved from independent horizontal gene transfer. Moreover, after 48-h incubation with chicken cecum *in vitro*, *Salmonella Heidelberg* was observed to acquire an IncK2 plasmid that carried an extended-spectrum-*β*-lactamase gene (*bla CMY-2*) (Oladeinde et al., 2019), demonstrating the possibility of resistant gene transfer *in vivo*.

### 2.3. Non-inherited resistance

Commonly, the susceptibility of bacteria to antimicrobial agents is quantified by MIC and MBC. Once the MIC is above the breakpoint, the strains are viewed as resistance. However, MIC and MBC aim at planktonic cells with normal growth rate. Unlike resistance (inherited, permanent), bacterial tolerance to antimicrobial agents is temporary and without mutations of resistant genes, which is commonly called non-inherited resistance or adaptive resistance (Fisher et al., 2017). The commonly discussed non-inherited resistance subpopulations of *Salmonella* are tolerant cells, persister cells, and biofilms. Among them, the form of tolerant cells and biofilms is commonly related to external stresses whose recognition may be mediated two-component systems (Murret-Labarthe et al., 2020). Given the similarity of above three phenotypes with inherited resistance, to help understanding the roles in combatting antimicrobial agents, their characteristics are concluded in Table 2 (Kim and Wood, 2017).

**Table 2:** The characteristics of *Salmonella* different phenotypes.

Phenotypes	Genetically	Under MIC of planktonic	Defense mechanism
Resistant	Inherited	Growth, alive	Genetically modified
Tolerant	Non-inherited	Slow growth, dead	Superoxide dismutase, RpoS
Persister cells	Non-inherited	No growth/no division, alive	Dormancy/low metabolism
Biofilms	Non-inherited	Slow growth/low metabolism, alive	Physical, hydrolyzed proteins

Owing to the slow growth rate, reduced metabolism level, low target activity, or low drug uptake of the non-inherited resistance subpopulations, the actual lethal or inhibitory effects of antimicrobial agents against them are significantly lower than that against their normal phenotype. S*almonella* non-inherited resistance is recognized that can cause antibiotic treatment failure, more frequent doses of antibiotics, and longer treatment duration and have been shown to lead to the higher resistant mutation frequency (Hall and Mah, 2017; Levin-Reisman et al., 2017). Here, we discuss recent progresses in understanding the producing mechanisms of *Salmonella* non-inherited resistance and provide the potential therapeutic strategies.

#### 2.3.1. Formation of biofilm

**Figure 2:**
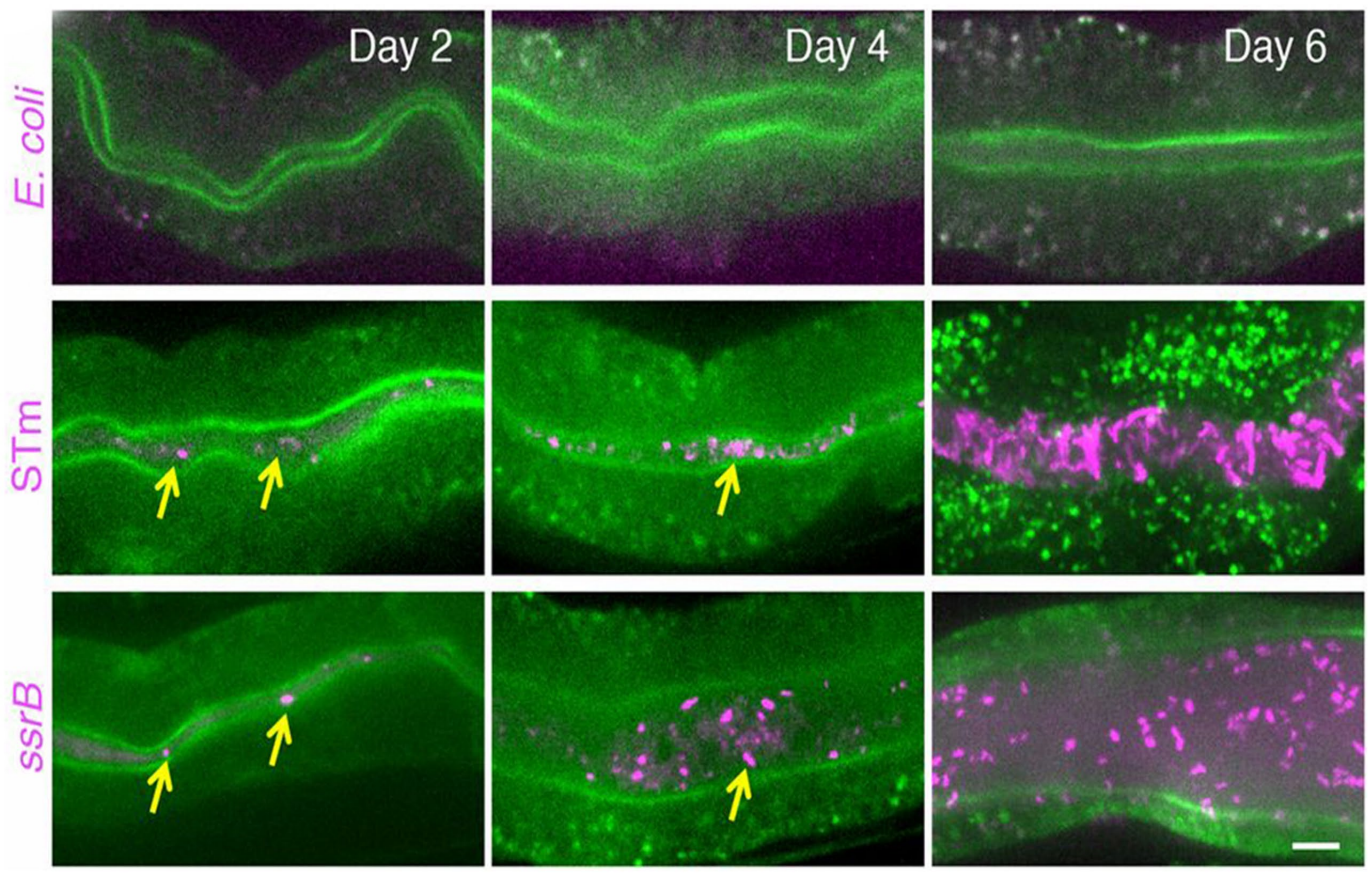
*Salmonella* forms biofilms within intestinal of *Caenorhabditis elegans*. Compared with *E. coli*, *Salmonella* tends to form biofilms *in vivo*. Reprinted with permission from Desai et al. (2019).

**Figure 3:**
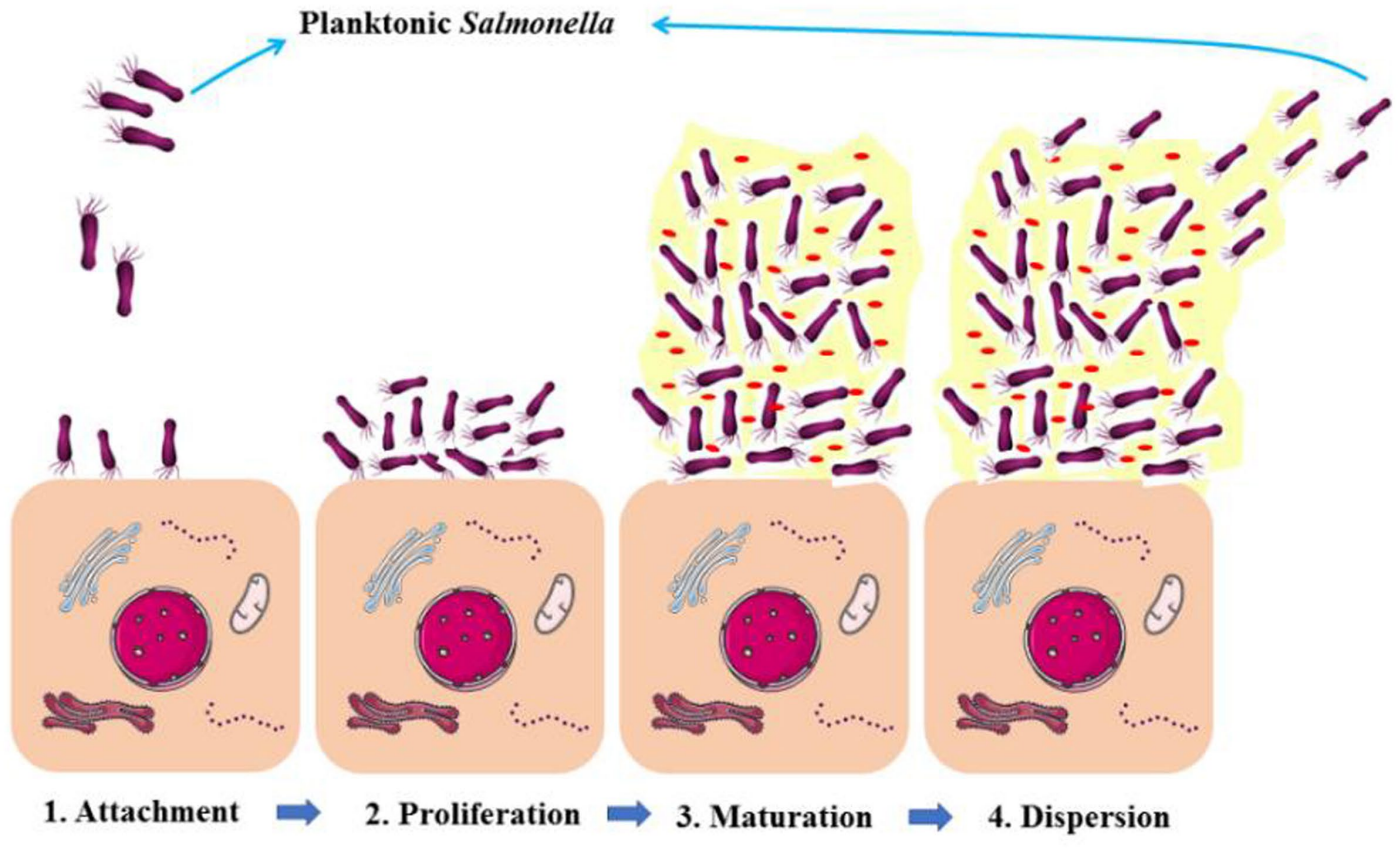
The schematic diagram stages of *Salmonella* biofilm formation. The forming of the biofilm contains four steps: (1) *Salmonella* attaches with the host cells; (2) proliferation; (3) biofilm maturation; (4) biofilm formed *Salmonella* disperse to planktonic *Salmonella*.

The ability of *Salmonella* to form biofilms *in vivo* was visualized by Desai et al. (2019). (Figure 2), which proved that *Salmonella* persistence and its asymptomatic carrier are closely related to its biofilms (Sandala et al., 2020). Reportedly, 2–5% of typhoid patients develop persistence and become asymptomatic carriers because of biofilm formation (Gunn et al., 2014). *Salmonella* biofilms are aggregates of *Salmonella* and its secretions, including extracellular DNA, exopolysaccharides, proteins, and metabolites, which are collectively referred to as extracellular polymeric substances (EPSs) (Das et al., 2013). After *Salmonella* attaches to the host cell surface, proliferation begins, and bacterial cells adhere to the surface irreversibly to form microcolonies. When enough individuals exist, *Salmonella* grows and matures from microcolonies into clusters of multilayered cells and begins to synthesize EPSs creating a biofilm. Meanwhile, some of cells within the biofilm will disperse in a planktonic state to continue to invade other host cells (Figure 3; Soto, 2013).

Due to the low permeability of antibiotics to biofilm matrix, the slow growth rate of the biofilms, the overexpression of efflux pumps, and the sheltering effects to persister cells (Lewis, 2010), *Salmonella* biofilms help the defense against antibiotics. Reportedly, González et al. (2018) found that *Salmonella* biofilms showed significantly higher MIC to ciprofloxacin both *in vitro* and *in vivo*. Moreover, 27 isolated *S. typhi* that formed biofilms displayed multidrug resistance (MDR) to doxycycline, sulfamethoxazole–trimethoprim, ciprofloxacin, ampicillin, and streptomycin (Shi et al., 2018). In addition, *Salmonella* can form biofilms on the surface of foods and food packaging, thus displaying resistance against disinfectants. For instance, peroxyacetic acid and acidified hypochlorite were not effective against biofilm-formed *Salmonella* compared with planktonic isolates (Chylkova et al., 2017). As mentioned above, food chain is an important transmission route of *Salmonella*, suggesting that an efficient tool for controlling *Salmonella* biofilms in poultry processing environments is urgently needed.

On the contrary, because of the nutrient limitation and oxygen gradients, the DNA oxidative damage of biofilms was in a high level (Hall and Mah, 2017). Therefore, *Salmonella* cells within biofilm show higher mutation frequency than that of planktonic cells (Hall and Mah, 2017). More importantly, unlike *Pseudomonas aeruginosa* and *Staphylococcus aureus*, *Salmonella* biofilm played a key role in ensuring activation of innate immunity rather than immunity escape. Desai et al.’s (2019) finding indicated that SsrB of *Salmonella* activated p38-MAPK innate immunity of *Caenorhabditis elegans*, allowing appropriate functioning of host innate immunity to eliminate the planktonic subpopulations, thus to confer itself an adaptive lifestyle advantage in persistent infections (e.g., asymptomatic carrying) because asymptomatic carriers are viewed as healthy population easily, which creates conditions for the wide spread of salmonellosis. Obviously, *Salmonella* biofilm status is particularly concerning in its persistent infections and pandemic (Desai et al., 2019). Therefore, the methods that can prevent the formation of *Salmonella* biofilms, in food chains and the clinical treatment, are worth further investigation.

#### 2.3.2. Persister cells

**Figure 4:**
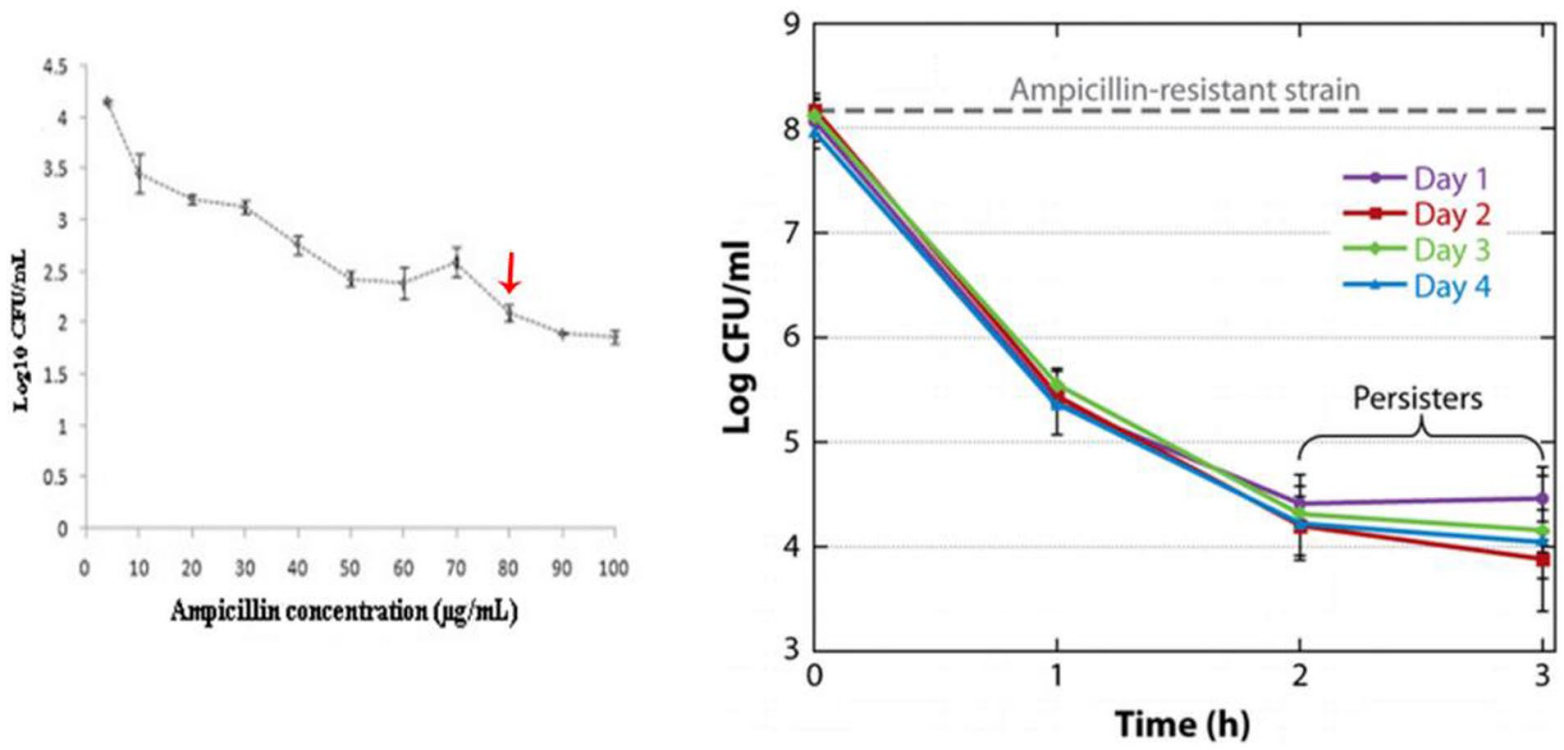
The tests for proving the presence of *Salmonella* persister cells. Reprinted with permission from Lewis (2010) and Rishi et al. (2018).

Another major tolerant strategy of *Salmonella* is the formation of persister cells, whose presence can be reflected by the killing curves. For instance, the more reduction in viability would not be recorded any more even under higher ampicillin concentrations or longer inhibition time (Figure 4; Lewis, 2010; Rishi et al., 2018). Persister cells not only are tolerant to high concentrations of antibiotics but can also undermine host immune defenses (Stapels et al., 2018). For instance, *Salmonella* persister cells reprogram macrophages by using its SPI 2 type 3 secretion system, in which the secreted effectors decreased proinflammatory innate immune responses and reduced anti-inflammatory macrophage polarization. Additionally, it was evidenced that *Salmonella* persister cells could help its acquired resistance in the gut (Bakkeren et al., 2019).

**Figure 5:**
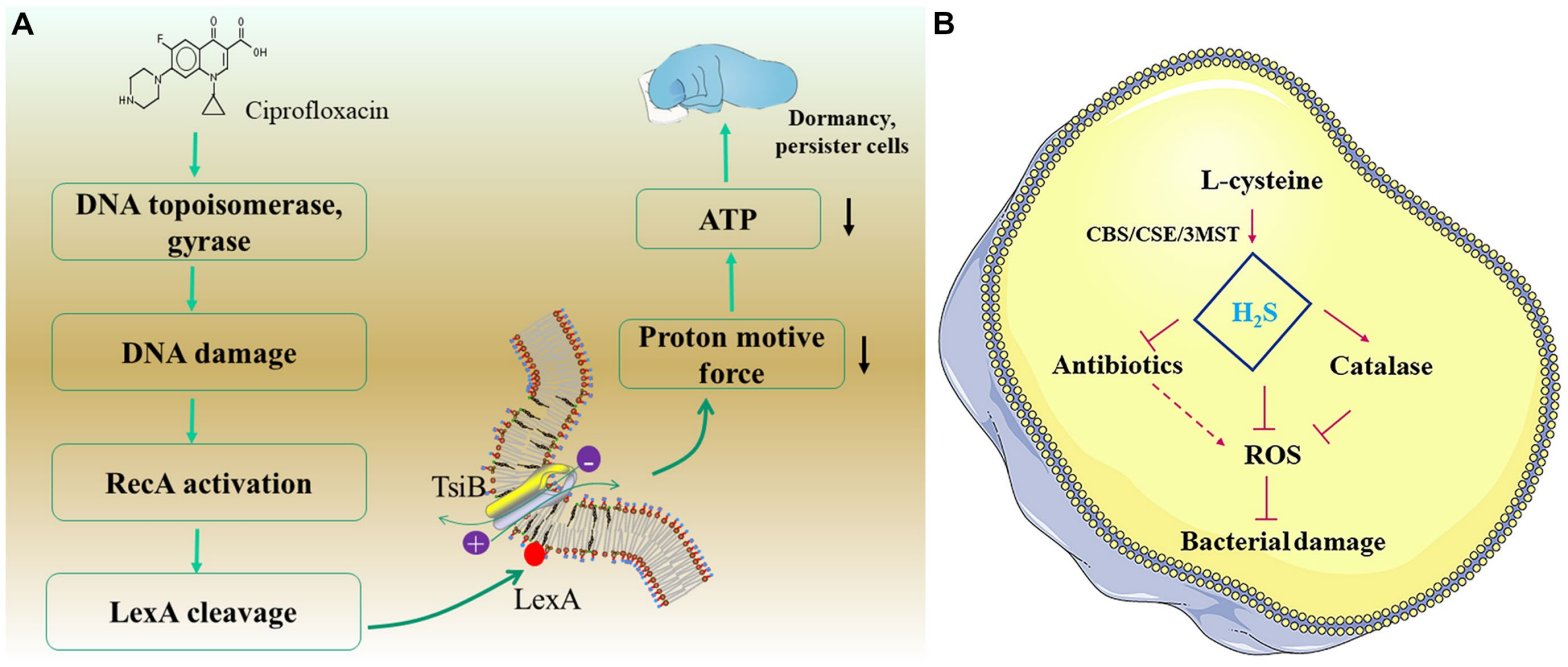
The two common strategies of the formation of persister cells. **(A)** The model of TisB protein-dependence. **(B)** The model of H_2_S-dependence.

Many excellent research revealed the mechanisms of the emergence of *Salmonella* persister cells, which could be divided into inhibition acetylation of aminoacyl-tRNAs, reduced ATP level, and increased H_2_S level. For instance, Rycroft et al. (2018) proved that the inhibiting acetylation of aminoacyl-tRNAs was achieved by blocking the primary amino group of the amino acid on the charged tRNA molecule with acetyltransferase. Additionally, Lewis (2010) proposed the mechanisms of the produce of *E. coli* persister cells under fluoroquinolones. When fluoroquinolones damage bacterial DNA, activated RecA will activate LexA repressor in turns, in which the increased TixB effector leads to a drop in proton motive force and ATP levels, causing the decrease in metabolism level of bacteria, thus promoting the formation of persister cells (Figure 5A). In view of the inhibition mechanisms of fluoroquinolones (aiming at DNA), we argued that the formation processes of *E. coli* persister cells under fluoroquinolones also meet *Salmonella*, which reminds us of the potential of metabolism and ATP promoter in combating *Salmonella* persister cells. After all, treating *Salmonella* with the ATP synthase poison arsenate caused the increase in persister cells (Braetz et al., 2017).

Interestingly, recent studies suggested that the emergence of H_2_S may be a kind of positive defense of bacteria, which helps bacteria into a mild poisoning state. Luhachack and Nudler (2014) proved that significantly higher H_2_S content was detected within the persister cells of *Pseudomonas aeruginosa* and *S. aureus* than their planktonic. Recently, oxidative stress has been proposed as one of the mechanisms whereby bactericidal antibiotics (e.g., fluoroquinolones and aminoglycosides) kill bacteria (Drlica and Zhao, 2021). H_2_S, the reductant within bacterial cells, protects bacteria from reactive oxygen species (ROS), thus to disrupt different classes of bactericidal drugs, especially to the bactericidal antibiotics (Figure 5B).

An undoubted issue is that *Salmonella* persister cells can resuscitate after removal of antibiotics, thus causing refractory infections. Furthermore, even when *Salmonella* exited the persister cell status, they still showed tolerance to the antibiotic, suggesting the presence of a long-retention effect or “memory effect” of persister cells (Miyaue et al., 2018). Additionally, *Salmonella* biofilm provides a shelter to the contained persister cells for evading immune response (Balaban et al., 2004).

Overall, there is no doubt that the biofilms and persister cells of *Salmonella* play a critical role in combating antibiotics and resistant evolution. However, during susceptibility tests, dose regimen design and clinical medication, biofilms, tolerance, and persistence are not put on a high level. At least, yet to date, no tolerance-targeting therapeutics have been reported from the Food and Drug Administration and WHO. Following the recent advances in combating *Salmonella* persister cells and biofilms, better medication strategies are discussed.

## 3. Overcoming *Salmonella* resistance by compounds

### 3.1. Enhancing permeation to OM

**Figure 6:**
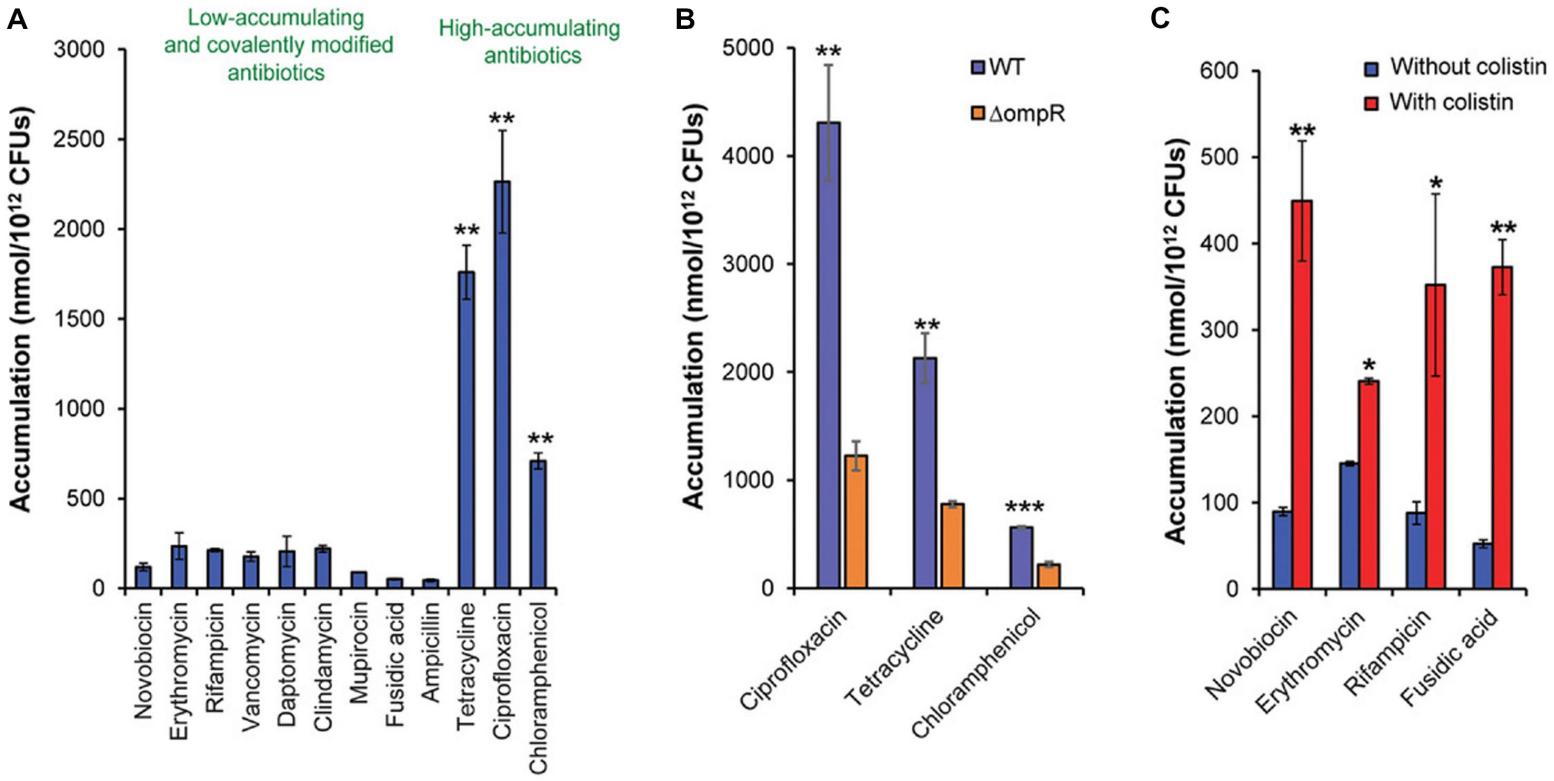
**(A)** The antibiotics with high accumulation within bacteria. **(B)** The roles of pore proteins in antibiotic permeation. **(C)** The enhancement efficiency of colisitin to permeation. Statistical significance was defined as *p < 0.05, **p < 0.01, and ***p < 0.001. **(A)** *p* values relative to the average of the low-accumulating controls; **(B)** *p* values relative to ΔompR in each group; **(C)** *p* values relative to without colistin in each group. Reprinted with permission from Richter and Hergenrother (2019).

As discussed above, due to the asymmetrical OM, in fact, only several antibiotics are permeable to the gram-negative pathogens. Although no specific studies focused the permeation of antibiotics against *Salmonella*, the studies on the validation of antibiotics accumulation in *E. coli* could be an indirect example that are helpful in enhancing penetration to *Salmonella* OM. As shown in Figure 6A, among different classes of antibiotics, only tetracycline, ciprofloxacin, and chloramphenicol showed ideal accumulation in *E. coli*. Once there was a lack of OmpR, the accumulation level of tetracycline, ciprofloxacin, and chloramphenicol was significantly reduced (Figure 6B). Meanwhile, combining with colistin, the accumulation level of antibiotics with low permeation was significantly increased (Figure 6C; Richter and Hergenrother, 2019).

The above results suggested that the two strategies could help the penetration of antibiotic against *Salmonella* OM: (a) possessing rational physicochemical properties and (b) combining with agents that aim at LPS. Many efforts have been paid in clarifying the correlation between accumulation in gram-negative pathogens and physicochemical properties of compounds. Physicochemical properties such as polarity hydrophobicity, molecular flexibility, and ring counts were recognized to be closely related to the cellular accumulation. Finally, the so called “eNTRy” rules were proposed. The small molecules possessed (a) ionizable nitrogen (primary > secondary > tertiary amines), (b) low three-dimensionality (globularity ≤0.25), and (c) relatively higher rigidity (rotatable bonds ≤5) which are most likely to accumulate in the bacterial cells (Prajapati et al., 2021). Additionally, as shown in Figure 6A, in view of the highest accumulation of ciprofloxacin (fluoroquinolones), we also guessed that -COOH might help in the penetration. The applying of these rules, such as in computer aid drug design, may promote the discovery of new compounds with high permeability to *Salmonella*.

In addition to design compounds rationally, the screening of MlaABC inhibitor agents is another potential strategy to increase the permeability of *Salmonella* OM. After all, bacterial strains with mutation of MlaABC system were evidenced to be more susceptibility. For instance, *E. coli* strains lack of MlaE or MlaD showed more susceptibility to EDTA and higher cellular accumulation of ethidium bromide or chlorpromazine (Tang et al., 2021). Furthermore, when clorobiocin, an inhibitor of MlaC, was combined with the human antimicrobial peptide LL-37, the antibacterial effects against *Acinetobacter baumannii* of the LL-37 were significantly improved (Huang et al., 2019). AA139, a synthesized peptide, targeting phospholipid transportation of bacteria, was evidenced to reverse the resistance of *Klebsiella pneumoniae*, *Pseudomonas aeruginosa*, *Acinetobacter baumannii*, and *E. coli*, while AA139 showed low spontaneous and induced resistance *in vitro* (Elliott et al., 2020), suggesting the huge potential of targeting MlaABC system in overcoming the *Salmonella* resistance.

### 3.2. Inhibiting efflux pumps

As mentioned above, efflux pumps of *Salmonella* play a critical role in reducing the cellular accumulation of antimicrobial agents, thus leading to its survival under high drug concentration. Inhibiting the activity of efflux pumps is an ideal strategy to enhance the efficiency of antimicrobial agents against *Salmonella*. For instance, Bonnefoy et al. (2004) provided a new *β*-lactamase inhibitor (AVE1330A), and when ceftazidime/AVE1330A was at a ratio of 4:1, only 2 μg/mL ceftazidime was required for *the* inhibition of resistant *Salmonella*. In addition, as mentioned above, macrolides were expelled from *Salmonella* cells by the MacAB efflux pump (Table 1), thus leading to *Salmonella* resistance to macrolides. Yamagishi et al. (2011) proved that the MacAB-expressing *Salmonella* strains were not sensitive to clarithromycin, azithromycin, leucomycin, or josamycin. However, after treatment with OU33858 (5-[(5-chloro-2-hydroxyphenyl) methylene]-3-propyl-2-thioxo-1,3-diazolidin-4-one, no activity against *Salmonella*), the growth of the MacAB-expressing *Salmonella* strains was clearly inhibited by the above macrolides, suggesting that OU33858 was an ideal MacAB efflux pump inhibitor. Grimsey et al. (2020) indicated that chlorpromazine and amitriptyline, substrates and inhibitors of the AcrB efflux pump, could reduce the AcrB efflux pump activity of *Salmonella*. It was reported that reserpine could be used as an AcrB efflux pump inhibitor of *Salmonella* (Shaheen et al., 2019). In addition, extracts of *Artemisia tournefortiana* could also reduce the expression of the *AcrB* efflux pump of *Salmonella* by downregulating the *acrB* gene in *S. enteritidis* strains (Khosravani et al., 2020).

More importantly, recent reports suggest that the EPIs show potency in limiting the horizontal transfer of resistance. Nolivos et al. (2019) found that although the resistance genes carried by plasmid could be transferred from donor bacteria to the recipient strains, the resistance protein could not be produced in the recipient strains without AcrB pump. By contrast, the resistance protein was produced in the wild-type strain. Therefore, Nolivos et al. (2019) highlighted that when we adopt the antibiotic treatment, if the inhibition method to AcrAB-TolC efflux pump was adopted at the same time, the horizontal transfer of resistance will be an uncommon issue. It suggests that the EPIs may have a second indirect effect: They can block the transfer of resistance between bacteria during antibiotic treatment, thus to limit the emergence of new resistant strains (Povolo and Ackermann, 2019). As a result, the screening of potential EPIs has got increasing attentions. According to the structure of EPIs, Lamut et al. (2019) summarized general structural characteristics of potential EPIs for us, for instance, containing at least two hydrophobic ring systems, the ring system can be quinolone, quinolone, benzene, pyridine, pyranopyridine, pyrimidine, pyridopyrimidinone, or indole.

### 3.3. Combatting non-inherited resistance

As mentioned above, in addition to resistant subpopulation, the biofilm and persister cell subpopulations of *Salmonella*, the non-normal growth or metabolism subpopulation, play a key role in combatting antibiotics and promoting resistant evolution. Consequently, the strategies on combating biofilms and persister cells are worth to be concluded.

Reportedly, Carvacrol and thymol inhibited biofilm formation of *Salmonella* at sub-MICs (Čabarkapa et al., 2019). Interestingly, in addition to the inhibition effect on efflux pump, efflux pump inhibitors (EPIs) were proved to possess the ability in inhibition to biofilm forming. Baugh et al. (2014) found that once the special inhibitor of AcrAB-TolC efflux pump (e.g., chlorpromazine, carbonyl cyanide m-chlorophenylhydrazone, or phenyl-arginine-naphthylamide) was inoculated with *Salmonella*, the biofilm forming was inhibited, by which they proposed that inhibition of efflux pump can be viewed as a strategy to prevent biofilm formation. Furthermore, Wang-Kan et al. (2017) indicated that when the *acrA*, *acrB*, or *tolC* gene (genes of AcrAB-TolC efflux pump) of *Salmonella* was deleted, compared with the wild-type *Salmonella*, the biofilm forming of mutant lacking strains was significantly reduced. Further analysis indicated that in mutants lacking *acrB* and *tolC*, the production of flagella genes was decreased, thus leading to the reduction of biofilm forming. As a reminder, the EPIs also possessed the inhibition ability to the biofilm of *Salmonella*. However, currently, there are few EPIs which can be used for medication.

On the contrary, the effects of antimicrobial agents targeting bacterial cell membrane compositions are less interfered by bacterial metabolic level and growth rate (Bahar and Ren, 2013; Rishi et al., 2018). For instance, Song et al. (2020) provided a short linear antibacterial peptide SLAP-S25, whose amino acid sequence was listed as CH_3_CO-Dab-Ile-Dab-Ile-Dab-dPhe-Leu-Dab-dVaL-Leu-Ala-NH_2_, which can be used to enhance the antibacterial activity of common antibiotics against MDR-resistant *Salmonella*. Mechanistic studies suggested that SLAP-S25 caused membrane damage by binding to both LPS and phosphatidylglycerol in the OM, thus allowing more antibiotics to enter the bacterial cells. As shown in Figure 4, the retention ~10^2^ CFU/mL bacteria are always observed in the antibiotic killing (inhibiting) curves, which is viewed as persister cells. The issue that we want to discuss is that the re-growth of the rest cells will cause reinfections. Compared with traditional killing curves, once the antibiotics were combined with SLAP-S25 (≤ 4 μg/mL), 100% inhibition rate could be achieved, suggesting that SLAP-S25 will be an efficient adjuvant to help most antibiotics to clear *Salmonella* persister cells.

The mechanism of persister cells show tolerance to high concentrations of antibiotics is commonly recognized as its low metabolism level (Brauner et al., 2016; Kim and Wood, 2017). Changing persister cells into the state of metabolically active and is recognized to be another strategy. For instance, Rishi et al. (2018) proved that mannitol displayed activity in increasing the metabolism level in the persister cells of *Salmonella*. When ampicillin was combined with mannitol, an approximately 78% elimination rate to the *Salmonella* persister cells was observed.

Notably, due to persister cells are few fraction of the total population, combining the adjuvants that aiming at persister cells with antibiotics not always can reduce the MIC value of antibiotics. For instance, Liu et al. (2021) found that indole-3-acetic acid (IAA) could not reduce the MIC of ciprofloxacin against MRSA *in vivo*, but the survival rate of the infected mice was significantly enhanced by the combining of IAA and ciprofloxacin. Further studies revealed that IAA enhanced the production of ATP and ROS within MRSA persister cells. As mentioned above, the decrease of ATP in bacteria is the classic characteristic of the emergence of persister cells under pressure of fluoroquinolones (Rishi et al., 2018), which reminds that IAA is an ideal adjuvant to deal with the recalcitrant infections of *Salmonella* caused by persister cells. What we want to highlight is that combining persister cell activators, the lower MIC will not be observed, but the better treatment efficacy *in vivo* can be achieved. As a reminder, if we always aspire to the reduction in MIC values, we will miss some valid metabolism activators for *Salmonella* persister cells.

**Figure 7:**
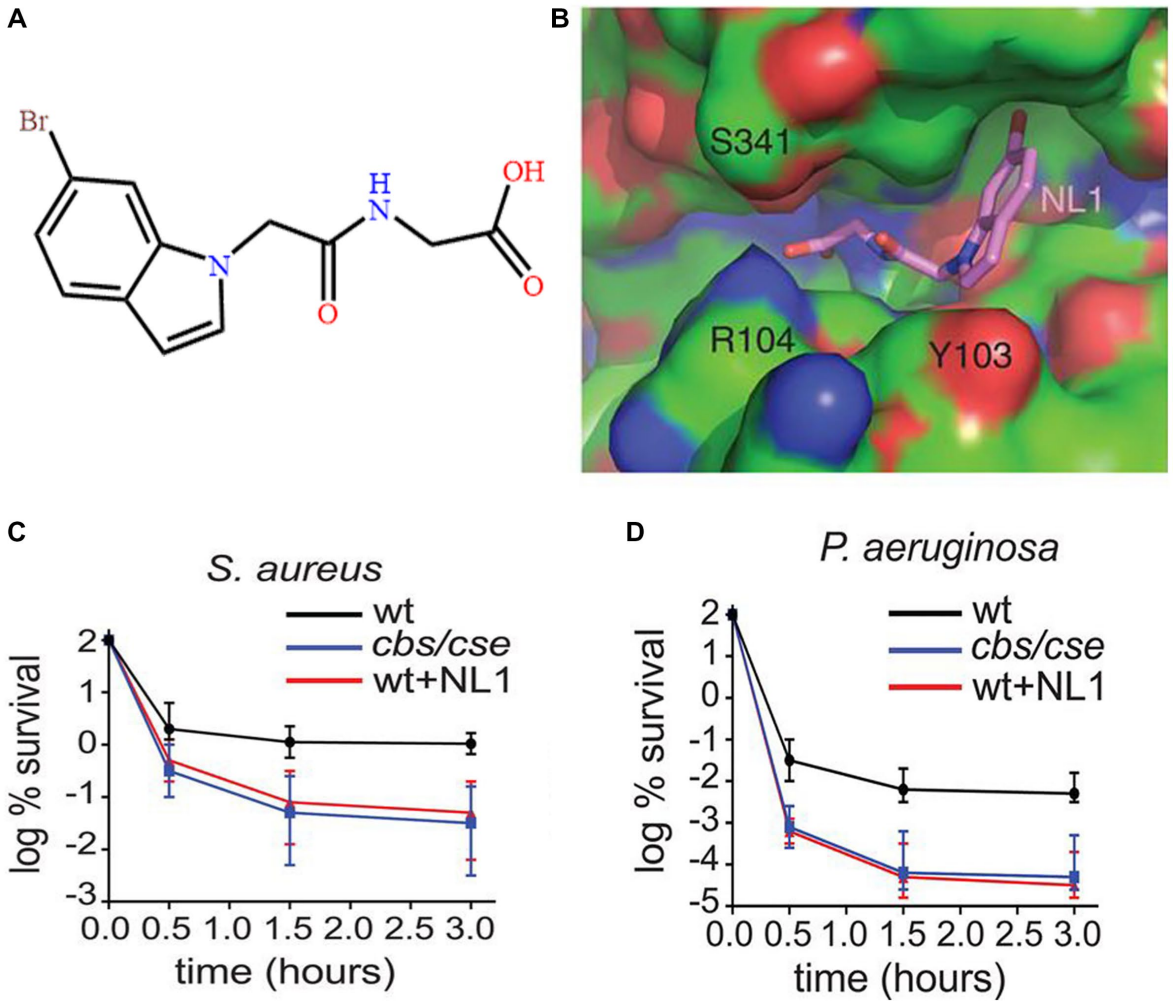
The effects of NL1 in reducing persister cells. **(A)** The construction of NL1. **(B)** View of NL1 (sticks) in the CSE binding pocket. **(C)** The effects of NL1 in reducing persister cells of *S. aureus*. **(D)** The effects of NL1 in reducing persister cells of *Pseudomonas aeruginosa*. Reprinted with permission from Shatalin et al. (2021).

On the other hand, to enter slight poisoning positively by producing low level H_2_S keep bacteria away from the damage of antibiotics. It reminded the potential of inhibitors aiming at H_2_S producing proteins. Shatalin et al. (2021) proposed a small molecule, NL1, which targeted CSE (Figure 7). As shown in Figures 7C,D, when NL1 was added, the counts of *Pseudomonas aeruginosa* and *S. aureus* persister cells were significantly reduced, and the survival rate of infection mice was improved by approximately 5-fold, which showed a brilliant prospect of the inhibitor agents targeting CSE in combatting persister cells of *Salmonella*.

## 4. Overcoming *Salmonella* resistance by bacteria

### 4.1. Probiotics

**Figure 8:**
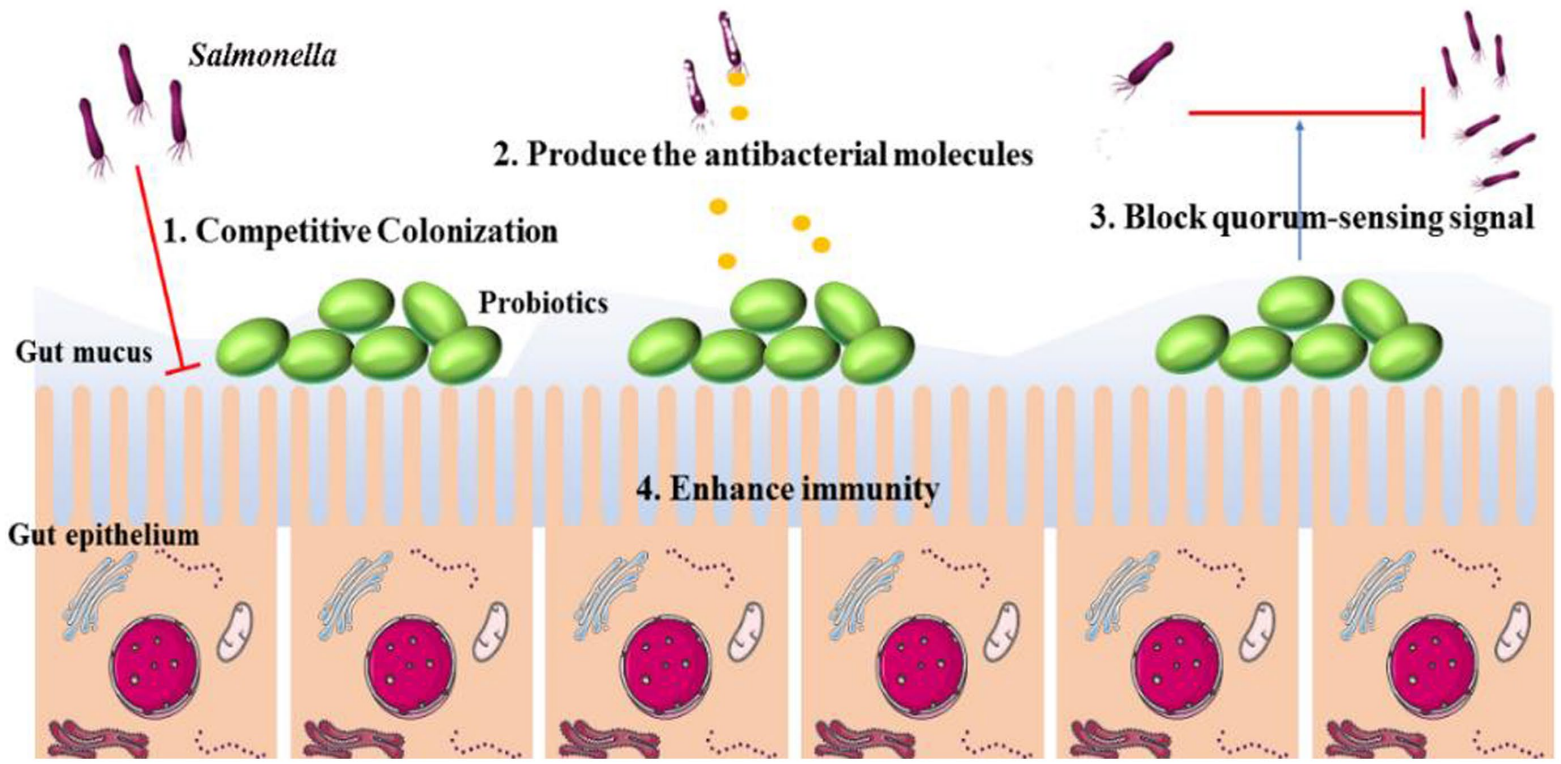
The mechanisms of intestine probiotic population prevent the colonization and invasion of Salmonella. The main mechanisms include: (1) competitive colonization; (2) produce the antibacterial molecules; (3) block quorum-sensing signal; (4) enhance host immunity.

The most proposed common groups of probiotics are *Lactobacilli* and *Bifidobacteria*. In addition, *E. coli*, *Saccharomyces cerevisiae* var., and *Bacillus coagulans* have been commercialized as probiotic products (Gut et al., 2018). As well known, the successful colonization of *Salmonella* is the first step of infections. Without colonization, further proliferation, biofilm formation, and intracellular survival will not occur. The main mechanisms by which probiotics can prevent resistant *Salmonella* infections were included as follows: (a) competitively inhibiting *Salmonella* colonization on the surface of host cells; (b) producing inhibitory agents to interfere with biofilm formation; (c) antibacterial molecules released by probiotics can kill resistant *Salmonella* directly; and (d) inhibiting resistant *Salmonella* by enhancing systemic immunity (Figure 8; Gut et al., 2018). Among the above mechanisms, competitive colonization inhibition is the most common. Although most studies do not focused on the elimination of resistant *Salmonella*, no evidence has shown that the colonization processes between resistant and sensitive *Salmonella* are different. The progresses of probiotics against *Salmonella* are concluded in Table 3.

**Table 3:** The examples of applying probiotics to treatment *Salmonella* infections.

Probiotic	Pathogen/animal models	Treatment mechanisms and outcomes	Reference
*L. casei*	1 day-old broiler chicks	Reduced colonization of chick’s gastrointestinal tract	Higgins et al. (2007)
FloraMax	Chicks and poultry	Reduced colonization	Menconi et al. (2011)
*Lactobacilli*	*S. typhi*	Secreted the molecules that can prevent cell invasion	Oelschlaeger (2010)
*L. plantarum*	*Salmonella*	Prevented intestinal colonization	Rokana et al. (2016)
*E. coli Nissle* 1917	Turkey	Lower carriage of *Salmonella* in the intestine	Forkus et al. (2017)

*L. casei*, *Lactobacillus casei*; *E. coli*, *Escherichia coli*; *B. lactis*, *Bifidobacterium lactis*; *L. plantarum*, *Lactobacillus plantarum*; *L. salivarius*, *Lactobacillus salivarius*.

**Figure 9:**
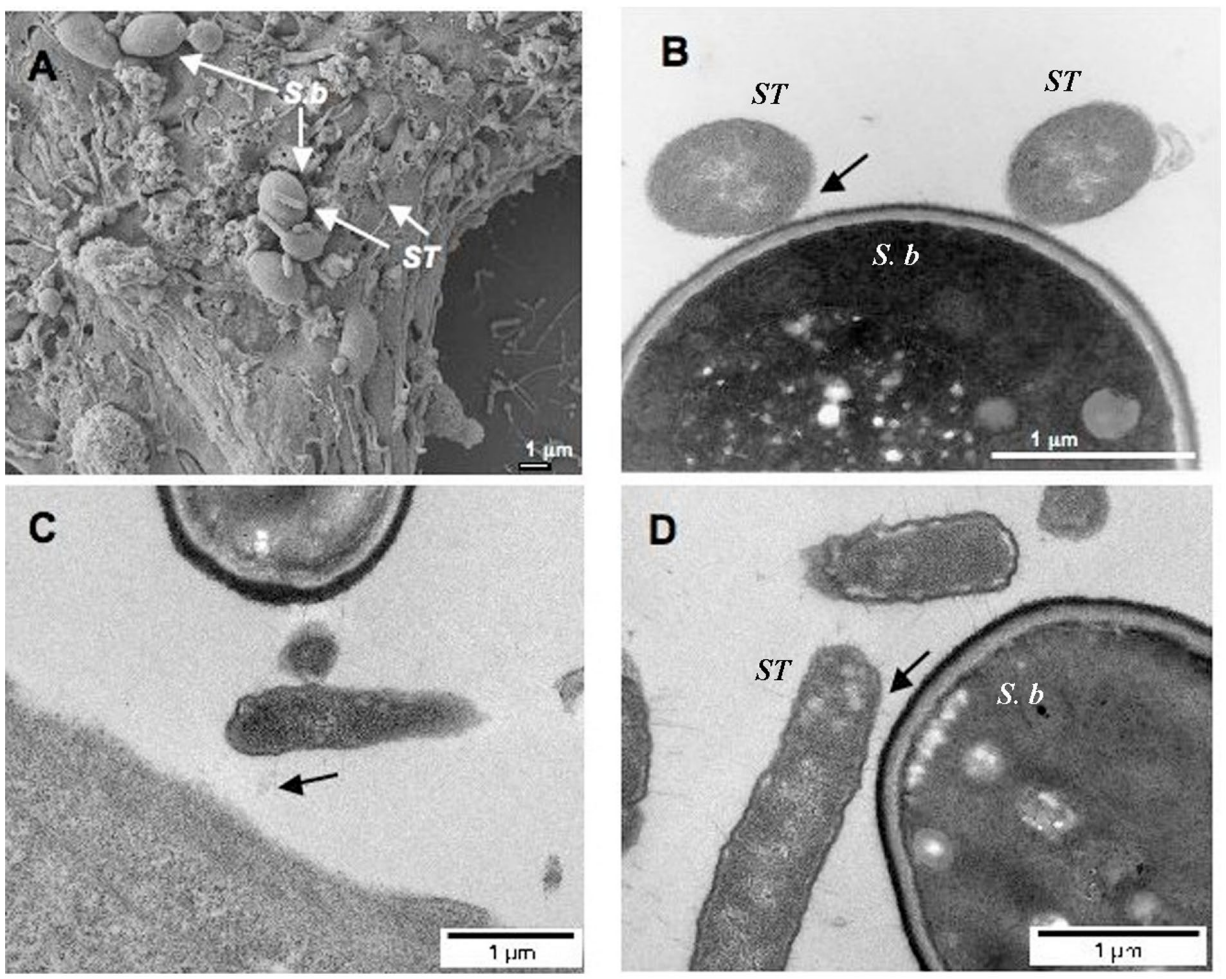
The imagines of *S. Typhimurium* (ST) binding to *S. boulardii* (Sb) cell wall. **(A)** Scanning electron micrograph of T84 cells infected by ST in the presence of Sb. **(B)** Transmission electron microscopy. **(C, D)** Transmission electron micrograph after red ruthenium staining. *N*=3. Black arrows show the binding of bacteria to the yeast. Reprinted with permission from Martins et al. (2010), licensed under CC-BY 4.0.

Previously, competitive colonization was thought to occur when the *Salmonella* colonization sites in the intestinal epithelium were occupied by probiotics. For instance, Vuotto et al. (2014) indicated that the attachment site of *Salmonella* on CaCo-2 cells was displaced by *Bifidobacterium longum* Bar 33. Recently, some studies reported that the adhesion of *Salmonella* on the surface of probiotics was one of the mechanisms to reduce *Salmonella* colonization. The close contact of *S. typhi* and *Saccharomyces boulardii* (*S. boulardii*) was confirmed by transmission electron microscopy (Figure 9; Martins et al., 2010). *S. typhi* colonized the gastrointestinal tract (GIT) of mice, but when the infected mice were administered *S. boulardii*, the bacterial cells clustered around the yeast cells, suggesting the adhesion action of *S. typhi* to *S. boulardii* (Pontier-Bres et al., 2014). Importantly, *S. boulardii* does not bind to the GIT, and after transitory retention in the GIT, the bound *Salmonella* will be excreted in the feces (Czerucka et al., 2007). It is speculated that the mechanism of binding is due to the presence of mannose-specific adhesion/receptors (fimbriae) on the cell wall of *Salmonella*, which can bind to mannose on the cell wall of yeast (Gut et al., 2018). Therefore, when yeast is used to prevent or control *Salmonella* infections, the intake of food- or beverage-rich sugars should be reduced.

Because of the natural antibiotic resistance and immunity escape, *Salmonella* biofilms are difficult to be cleared. It was reported that several enhancement mechanisms are associated with the inhibition of probiotics against biofilm formation. One of the mechanisms is that probiotics can produce enzymes to interfering *Salmonella* biofilm. Alpha-amylase, an enzyme produced by yeast cells, was reported to prevent biofilm formation of *Salmonella* (Sadekuzzaman et al., 2018). Moreover, the biomolecules produced by probiotics could destroy the construction of the bacterial biofilm. For instance, the exopolysaccharide produced by *Lactobacillus* spp. showed inhibitory activity against biofilms of *Salmonella* (Xu et al., 2020). Additionally, lectin-like protein (Llp) 1 and Llp2 produced by *Lactobacillus rhamnosus* GG showed pore-like action on *Salmonella* biofilms. More interestingly, neither Llp1 nor Llp2 could prevent biofilm formation of other pathogens, suggesting that the Llp1 and Llp2 lectins may have the *Salmonella*-specific activity (Petrova et al., 2016)^.^

Although the probiotic method, which treats *Salmonella* with bacteria, showed exciting in combating resistant *Salmonella*, there are few commercial probiotic products that are used for the treatment of *Salmonella* infections (Ghosh et al., 2019). One of the reasons is the lower treatment efficacy. For instance, the results of probiotic feeding indicated that probiotics had the potential to enhance the luminal microbiota; however, the amount of *S. typhi* found in the feces was not significantly reduced (Khan and Chousalkar, 2019). On the contrary, it is not easy to screen an activity probiotic against *Salmonella*. To simplify the screening processes of efficient probiotics, fecal transplant treatment (FTT) technology that transplants the microbiome from a healthy donor into a diseased gut is an alternative strategy. Because efficient probiotics are contained in the feces of healthy donors, FTT therapy has shown promise in clearing colonization with multidrug-resistant Enterobacteriaceae such as *E. coli*, *Salmonella*, and methicillin-resistant *S. aureus* (Piewngam et al., 2018), especially for the treatment of *Clostridium difficile* infections. However, few studies of FTT against *Salmonella* infections in humans and in veterinary clinics are reported. We argue that the FTT method is a potential breaker to combat resistant *Salmonella*. At least in the veterinary clinic, the feces of healthy animals can be mixed with feed to control animals’ *Salmonella* infections.

### 4.2. Predatory bacteria

**Figure 10:**
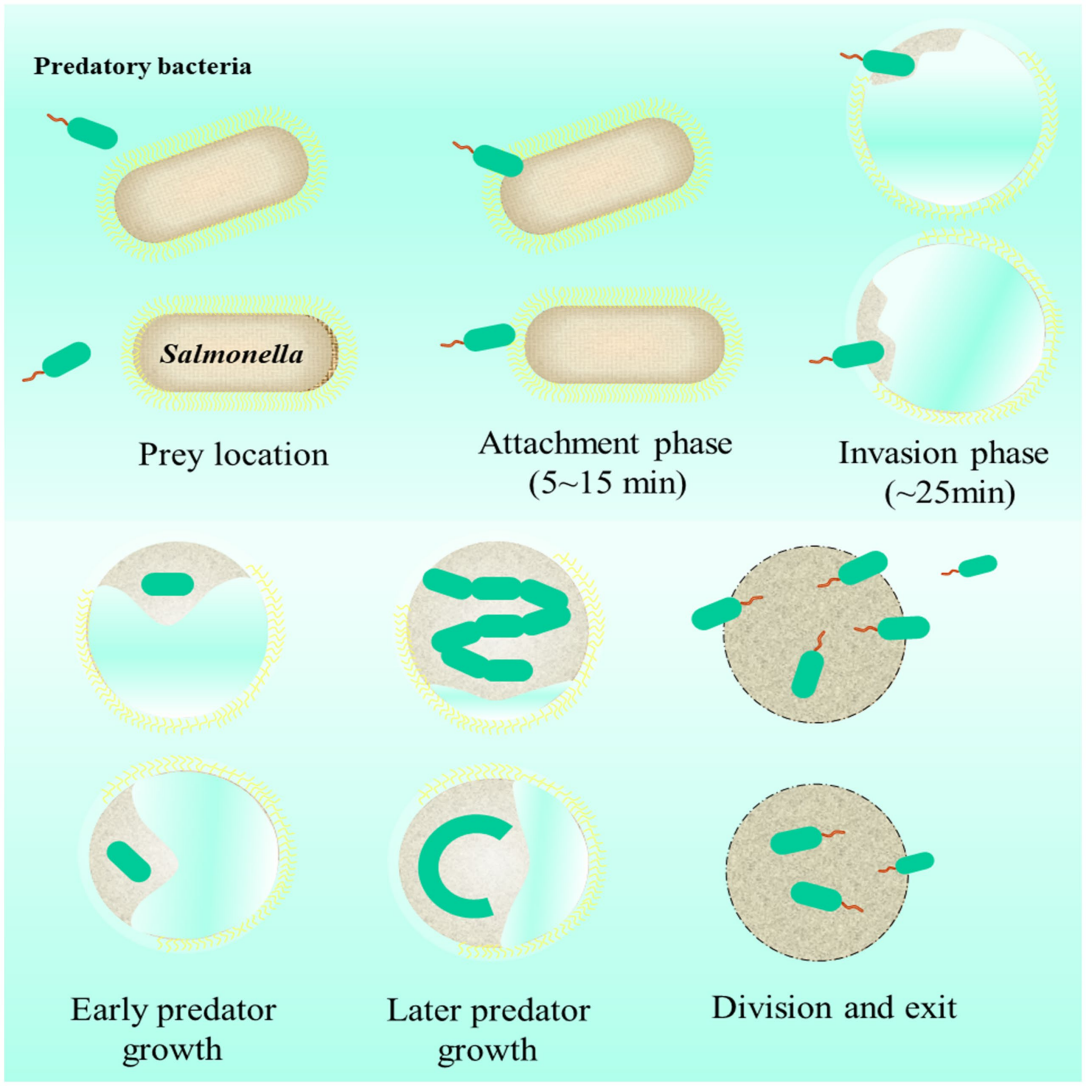
The life cycle of predatory bacteria attacking *Salmonella*.

Predatory bacteria are commonly called “killer of gram-negative bacteria.” Being different from phages, they multiply only after entering gram-negative pathogens (e.g., *E. coli*, *Salmonella*) (Kadouri et al., 2013). Common predatory bacteria are *Bdellovibrio* and *Bdellovibrio-*like organisms, such as *Bdellovibrio bacteriovorus* and *Micavibrio aeruginosavorus* (Negus et al., 2017). The invasion processes and life cycle of *Bdellovibrio bacteriovorus* in gram-negative are displayed in Figure 10 (Sockett and Lambert, 2004; Cavallo et al., 2021). During predation, predatory bacteria seek for prey by flagellum propelling. Once preys are caught, the location between predatory bacteria with prey occurs. During attachment, predatory bacteria swim at high speeds (~160 μm/s) to penetrate a prey cell. Then, predatory bacteria establish themselves within the prey cell periplasm by remodeling the wall and attaching to the cytoplasmic membrane of the prey cells. After that, predatory bacteria produce several progeny cells by using the nutrients within prey cells. Finally, when nutrients within prey cell are exhausted, the division cells format flagellum to exit old prey cells and start to seek for further prey.

Due to its specificity and efficiency, prey ability to gram-negative, predatory bacteria is called “alive antibiotics” (Cavallo et al., 2021). The studies on using predatory bacteria to combat *Salmonella* are excited. Atterbury et al. (2011) proved that after oral administration of *Bdellovibrio*, the amount of *S. enteritidis* in the chicken gut was significantly reduced. As mentioned above, during invasion, predatory bacteria can form a local pore on the cell wall of bacterial pathogens, suggesting that the enhancement of antibiotic intracellular delivery could be achieved by combining predatory bacteria with antibiotics. Shatzkes et al. (2017) proved that the antibacterial activity of predatory bacteria against resistant *E. coli* was the same as that against sensitive *E. coli*, which also demonstrated the antibacterial potential of predatory bacteria against resistant *Salmonella*.

As mentioned above, the cell wall damaging agents are one of the potential measures to combat the *Salmonella*-tolerant cells. Being different from most antibiotics, the predation ability (bactericidal capacity) of predatory bacteria is not limited by the growth state of *Salmonella*, that is, when predatory bacteria are applied to treat salmonellosis, the few tolerant cell formation can be expected. For instance, the enzymes produced by predatory bacteria can help the penetration to bacterial biofilms (Wucher et al., 2021). Importantly, this reminds the advantage of predatory bacteria in eliminating *Salmonella* biofilms attach on the surface of food (Sun et al., 2017).

As one of the foodborne pathogens, animal-derived food (e.g., meat, milk, and egg) is a common route of *Salmonella* transmission. Meanwhile, due to the presence of co-resistance between disinfectants with antibiotics, the use of disinfectants in environment promotes the development of *Salmonella* resistance. The application potential of predatory bacteria being used as a biology disinfection agent is obtaining more and more attention. Waso et al. (2021) proposed that compared with using chemistry disinfectants and antiseptics, the predatory bacteria method shows more superiority in limiting the emergence of co-resistance against disinfectants and *Salmonella* and reducing biofilms, suggesting the application potential of predatory bacteria in food packaging and environmental disinfection.

As mentioned above, one of the important resistance mechanisms of *Salmonella* is that it can obtain resistance genes by horizontal spread, which is called acquired resistance. Fortunately, because of its kinds of enzymes (e.g., nuclease), predatory bacteria showed enormous potential in the degradation of antibiotics resistant genes (Bratanis et al., 2017; Bratanis and Lood, 2019). As a reminder, if predatory bacteria were used to treat *Salmonella* infections, not only could the resistance *Salmonella* be killed, but also the horizontal spread of resistant genes could be limited. As proposed by Sockett and Lambert (2004), due to the poor segregation during the co-encytic multichromosomal bdelloplast stage and highly motile as well as fast-swimming, one of the native characteristics of predatory bacteria is that they can reduce the opportunities for conjugative transfer of resistant genes from other bacteria species.

Reportedly, *Bdellovibrio* was isolated from mammalian feces, suggesting the probable presence of predatory bacteria in the mammalian intestine (Schwudke et al., 2001), that is, predatory bacteria are probable safe to mammalian. However, studies on predatory bacteria are very limited. Their biosafety remains a concern that needs further evaluation.

## 5. Concluding remarks and further prospects

*Salmonella* infections severely threaten human health, and increasing antibiotic resistance continuously leads to a worsening situation. The antibiotic resistance of *Salmonella* is multifactorial in that it can occur through intrinsic (OM limitation, efflux pumps and antibiotic-inactivating enzymes) and acquired (mutations and acquisition of resistance genes) mechanisms. Meanwhile, biofilms and persister cells of *Salmonella* play a critical role in antimicrobial agent escaping, resistant evolution, and pandemic. Due to the strong abilities of *Salmonella* in combating antimicrobial agents, traditional antibiotic therapy is difficult to control its spread and resistant development. To solve the challenges caused by *Salmonella* resistance, this review included some alternative therapeutic strategies that have the potential to combat the resistance of *Salmonella*.

However, most of strategies only are validated *in vitro*. Regardless of small compounds, polypeptide, probiotics, or predatory bacteria, only a suitable dose could have enough antibacterial effect on resistant *Salmonella in vivo*. Therefore, how to design a suitable dose is worth to be discussed. Only then can these potential resistance breakers be safely applied in the clinic. Because of the large experimental animal uses in conventional PK-PD methods during dose design, the more economical hollow fiber model has received increasing attention (Ferro et al., 2015), which has been qualified by the European Medicines Agency as a methodology for use in support of selection and development of anti-tuberculosis regimens (Cavaleri and Manolis, 2015) Reportedly, the acquired resistance degrees of MDR *Mycobacterium tuberculosis* under different doses of levofloxacin were evaluated by a hollow fiber model; thus, the optimized antibacterial and prevention mutation dose of levofloxacin was provided (Arber, 2014), suggesting that the treatment efficacy of antibiotics plus antibiotics and antibiotics plus adjuvants against *Salmonella* could also be studied by the hollow fiber infection model (Srivastava et al., 2017). Furthermore, even the efficient inoculation number of probiotics and predatory bacteria can also be designed via a hollow fiber infection model by monitoring the number of *Salmonella* versus time (D’ambrosio et al., 2022).

**Figure 11:**
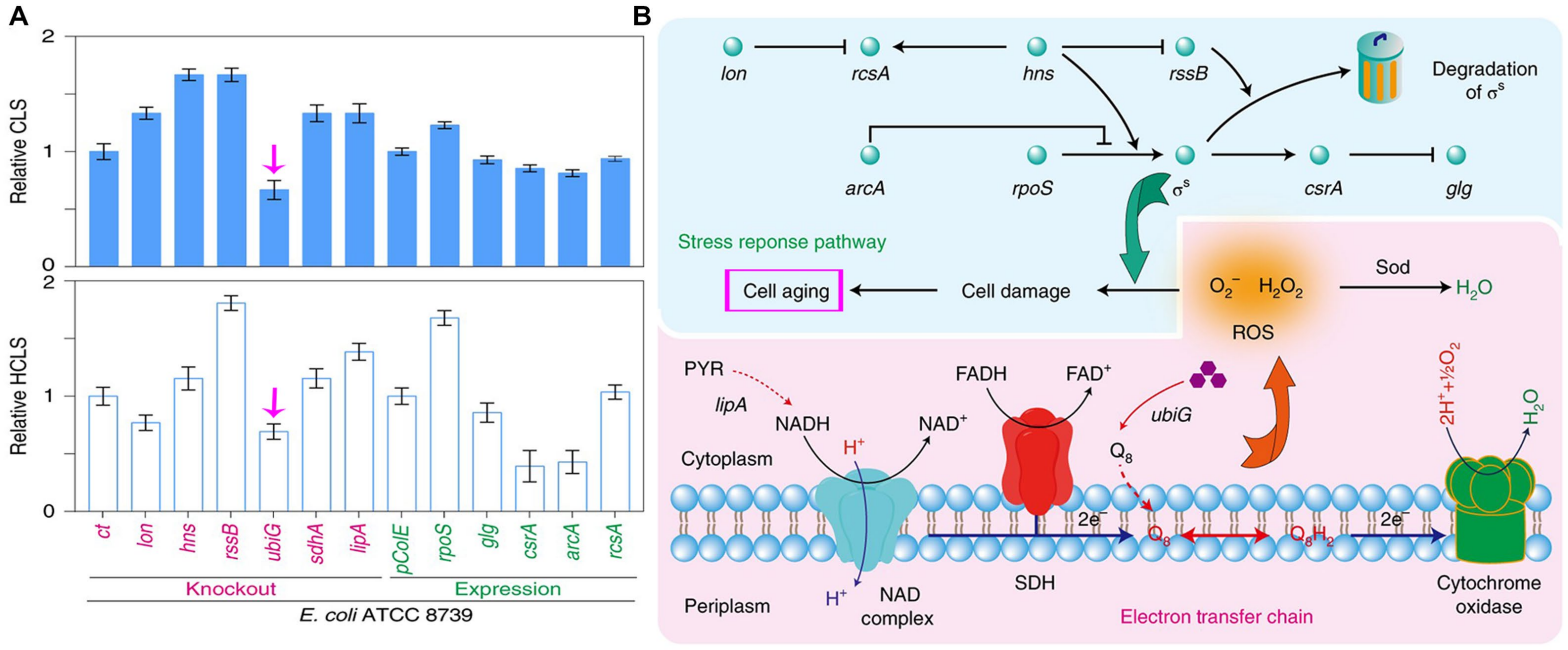
Regulating chronological lifespan (CLS) of bacteria by genetic manipulation. **(A)** Knockout *ubiG* gene of *E. coli* could short its CLS and half-chronological lifespan (HCLS). **(B)** The modulation of probiotics or predator bacteria lifespan may be achieved by regulating the electron transfer chain and stress response pathways. Reprinted with permission from Guo et al. (2022), authorized by Nat. Catal.

More important issues of probiotics and predatory bacteria are their retention *in vivo* and potential biological risks. After probiotics and predatory bacteria being used for treatment, how to elimination them from body is a widely concerned problem (Ghosh et al., 2019). Recent report indicated that chronological lifespan (CLS) of *E. coli* could be shorted 33% by deleting the methyltransferase gene (*ubiG*) (Figure 11A), while the regulation was evidenced to be a robust, strain-independent regulation (Guo et al., 2022). This suggests that the CLS of probiotics and predatory bacteria may also be controlled (Figure 11B). In this case, once the treatment time required for *Salmonella* infections is met, probiotics or predatory are automatic death *in vivo*. By this way, the potential biological risks and resistance spread risks of probiotics and predatory will not be an issue any more.

Finally, as proved by Liu et al. (2021), persister cells as a small part of population, combining antibiotics with persister cells inhibitors not always causes a lower MIC value, but better treatment efficacy *in vivo* can be achieved. We want to highlight is that the goal during antibiotic chemotherapy is the cure of infections, and the traditional view only focusing on the MIC decrease *in vitro* is the time to be changed. After all, the cure rates are not only related to the susceptibility of bacterial pathogens to antibiotics but also the presence of the non-inherited phenotype (Zappala, 2004; Shatalin et al., 2021).

## Author contributions

KZ and LH: conceptualization. XZ, LS, XX, and WM: methodology. KM, LS, XZ, XX, and WM: investigation. KZ: writing—original draft. KZ and LH: writing—reviewing and editing. LH: funding acquisition and supervision. All authors have read and agreed to the published version of the manuscript.

## Funding

This research was funded by the National key research and development program (2022YFD1800402) and the National Natural Science Foundation of China (32273063).

## Conflict of interest

The authors declare that the research was conducted in the absence of any commercial or financial relationships that could be construed as a potential conflict of interest.

## Publisher’s note

All claims expressed in this article are solely those of the authors and do not necessarily represent those of their affiliated organizations, or those of the publisher, the editors and the reviewers. Any product that may be evaluated in this article, or claim that may be made by its manufacturer, is not guaranteed or endorsed by the publisher.
